# Involvement of the *bla*_CTX-M-3_ gene in emergence of a peculiar resistance phenotype in *Klebsiella pneumoniae*

**DOI:** 10.3389/fcimb.2025.1545157

**Published:** 2025-05-13

**Authors:** Peishan Li, Leping Yan, Jingjie Song, Chengfeng Lin, Fangyin Zeng, Shihan Zeng

**Affiliations:** Department of Clinical Laboratory, Fifth Affiliated Hospital, Southern Medical University, Guangzhou, China

**Keywords:** CTX-M-3, *Klebsiella pneumoniae*, peculiar resistance phenotype, cefepime, ceftazidime

## Abstract

**Introduction:**

This study aimed to investigate the mechanism underlying a peculiar resistance phenotype in *Klebsiella pneumoniae*, characterized by reduced susceptibility to cefepime compared to ceftazidime.

**Methods:**

Antimicrobial susceptibility testing, plasmid conjugation experiments, whole-genome sequencing, and bioinformatic analyses were employed to characterize the resistance phenotype and identify genetic determinants.

**Results:**

A total of 20 *K. pneumoniae* strains exhibiting peculiar resistance phenotypes were collected and analyzed. Ten distinct sequence types (STs) were identified, including ST25 (4/20), ST967 (3/20), ST65 (2/20), ST133 (2/20), ST48 (2/20), ST353 (1/20), ST628 (1/20), ST753 (1/20), ST792 (1/20), and ST254 (1/20). All strains were resistant to FEP (MIC_50_ = 128 µg/mL) but not to CAZ (MIC_50_ = 8 µg/mL). This resistance was primarily attributed to the presence of the *bla*_CTX-M-3_ (14/20) and *bla*_OXA-10_ (3/20). Conjugation experiments demonstrated that 5 out of 14 *bla*_CTX-M-3_-positive *K. pneumoniae* strains successfully acquired transconjugants, which exhibited the same peculiar resistance phenotype. PCR analysis confirmed that the conjugates contained the IncFII plasmid. To further elucidate the genetic basis of the resistance phenotype, whole-genome long-read sequencing was performed on three *bla*_CTX-M-3_-positive *K. pneumoniae* strains. The sequencing results confirmed that *bla*_CTX-M-3_ was located on the IncFII plasmid, and analysis of its genetic environment revealed a frequent association with mobile genetic elements such as IS*26*, IS*Ecp1*, and Tn*3*.

**Discussion:**

The primary driver of this phenotype in *K. pneumoniae* is the presence of the IncFII plasmid carrying *bla*_CTX-M-3_, which contrasts with the resistance mechanisms often reported in *Pseudomonas aeruginosa* exhibiting similar phenotypes. This study emphasizes the critical role of plasmid-mediated resistance in the spread of multidrug resistance in *K. pneumoniae* and provides insights into strategies for combating resistance in these pathogens.

## Introduction

*Klebsiella pneumoniae* (*K. pneumoniae*) is a significant Gram-negative bacillus (GNB) responsible for nosocomial infections, including pneumonia, urinary tract infection, and bloodstream infections (Munoz-Price et al., 2013). The rise in multidrug-resistant strains of *K. pneumoniae* has heightened concerns in clinical environments, complicating treatment protocols and contributing to increased morbidity and mortality (Liu et al., 2022). Cephalosporins, particularly third and fourth-generation agents, are widely used for treating *K. pneumoniae* infections due to their broad-spectrum efficacy. However, certain *K. pneumoniae* strains have demonstrated a peculiar resistance phenotype, showing reduced susceptibility to the fourth-generation cephalosporin cefepime (FEP) compared to the third-generation cephalosporin ceftazidime (CAZ). This phenotype is consistently referred to as the “peculiar resistance phenotype” throughout the article. Importantly, the clinical significance of this resistance phenotype seems to be largely overlooked. This atypical resistance pattern poses a clinical challenge, as FEP is generally considered more potent than CAZ against resistant strains.

FEP exhibits potent antibacterial activity against GNB, including *K. pneumoniae* and *Escherichia coli* from the *Enterobacteriaceae* family, as well as non-fermentative bacteria such as *Pseudomonas aeruginosa* (*P. aeruginosa*) *(* Okamoto et al., 1994; Nguyen et al., 2014; Rodriguez-Bano et al., 2018). Notably, a review of the literature indicates that research on this peculiar resistance phenotype primarily focuses on *P. aeruginosa*, with limited studies addressing other bacterial species. Mechanisms such as the overexpression of the mexXY-OprM efflux system and the emergence of various β-lactamases, including *bla*_OXA-1_, *bla*_OXA-10_, *bla*_OXA-31_, *bla*_OXA-35_, and *bla*_PSE-1_, have been identified as significant contributors (Aubert et al., 2001; Hocquet et al., 2006; Campo Esquisabel et al., 2011). These mechanisms enable the bacteria to evade the β-lactam antibiotics, leading to treatment failures. Although *K. pneumoniae* is one of the common pathogenic GNB, reports detailing the mechanisms underlying this peculiar resistant phenotype remain limited. This raises the question of whether there are differences in resistance mechanisms between *K. pneumoniae* and *P. aeruginosa*. This gap in the literature underscores a critical need for further investigation into the genetic and biochemical pathways involved. Understanding these mechanisms is crucial for developing effective therapeutic strategies to combat the rising threat of antibiotic resistance in *K. pneumoniae*.

## Materials and methods

### Bacterial strains

Between January 1, 2023, and June 30, 2023, peculiar resistance phenotypes of *K. pneumoniae* strains were isolated from patient specimens at the Fifth Affiliated Hospital of Southern Medical University, China. To ensure the integrity of the subsequent research, a total of 20 unique *K. pneumoniae* were selected after removing duplicates. All strains were identified using matrix-assisted laser desorption/ionization time-of-flight mass spectrometry (MALDI-TOF MS) (Vitek MS; bioMérieux, France).

### Antimicrobial susceptibility testing

The minimum inhibitory concentration (MIC) values for FEP, CAZ, amikacin (AMK), amoxicillin-clavulanic acid (AMC), ertapenem (ETP), cotrimoxazole (SXT), piperacillin-tazobactam (TZP), tigecycline (TGC), cefuroxime (CXM), cefuroxime axetil (CXA), ceftriaxone (CRO), cefoxitin (FOX), imipenem (IPM), and levofloxacin (LVX) were performed using VITEK-2 Compact equipment. To precisely determine the MICs of CAZ and FEP, broth microdilution assays were performed in sterile 96-well plates. Twofold serial dilutions of antimicrobial agents were prepared in Mueller-Hinton broth (MHB) with the following concentration ranges: FEP from 1,024 to 0.0625 μg/mL and CAZ from 16 to 0.0625 μg/mL. Bacterial suspensions were standardized to 0.5 McFarland units (~1×10⁸ CFU/mL) and diluted 1:100 in MHB prior to inoculation. Each plate included growth controls (broth + inoculum without antibiotics) and sterility controls (broth only). Plates were incubated aerobically at 35°C for 18–20 hr before visual MIC determination. All procedures and result interpretations followed the Clinical and Laboratory Standards Institute (CLSI) guidelines, CLSI M100-2024.

### Plasmid conjugation experiments

The transferability of plasmids carrying resistance-related genes was determined by the conjugation experiments using *K. pneumoniae* exhibiting peculiar resistance phenotype as donors and rifampin-resistant *E. coli* C600 as a recipient. Transconjugants were selected on Luria-Bertani agar plates supplemented with rifampin (100 µg/mL) and FEP (4 µg/mL). PCR sequencing (refer to **Supplementary Table S1** for the relevant primer sequences) was employed to verify the presence of the *bla*_CTX-M-3_ gene in the transconjugants. Subsequently, antimicrobial susceptibility testing was conducted to confirm the antimicrobial resistance profiles of these transconjugants.

### Whole-genome sequencing and bioinformatics analysis

Genomic DNA from *K. pneumoniae* isolates exhibiting peculiar resistance phenotype was extracted using a bacterial genomic DNA extraction kit (Tiangen, Beijing, China) and subsequently sequenced on the Illumina NovaSeq 6000 platform. The original clean offline sequences were assembled with SPAdes 3.13.1 and annotated using Prokka 1.14.5 (Bankevich et al., 2012; Seemann, 2014). Sequence types (ST) were determined through multilocus sequence typing (MLST 2.18.0). The resistance genes and plasmid replicon types of the isolates were analyzed using the ResFinder 4.1 and PlasmidFinder 2.1 web tools from the Center for Genomic Epidemiology (CGE), applying default thresholds of a minimum identity percentage of 90% and a minimum coverage percentage of 60% (Bortolaia et al., 2020). Five representative *K. pneumoniae* isolates exhibiting peculiar resistance phenotype were selected for sequencing on the Oxford Nanopore platform. A mixed genome assembly of long and short reads was conducted using Unicycler 0.4.8 (Wick et al., 2017). The various plasmid backbones carrying associated resistance genes were visualized through BRIG analysis, while the genetic context of different related resistance genes was illustrated using Easyfig (Alikhan et al., 2011; Sullivan et al., 2011). Additional genomic sequences of *K. pneumoniae* carrying *bla*_CTX-M-3_ gene were retrieved from the NCBI database and used for phylogenetic analysis alongside the strains sequenced in this study. Core-genome SNPs were extracted using Snippy v4.6.0 (https://github.com/tseemann/snippy). IQ-TREE2 v2.3.6 (https://github.com/iqtree/iqtree2) was then used to construct the phylogenetic tree with the TVM+F+ASC+R5 model.

### Nucleotide sequence accession number

This Whole Genome Shotgun project has been deposited in the DDBJ/ENA/GenBank under the accession IDs: JBJFKS000000000 to JBJFLL000000000. This range represents a collection of 20 genome sequences, each assigned a unique accession number. The individual genome data can be accessed through the respective accession numbers. The complete nucleotide sequences of plasmids plncFll+FIB_OXA10-217-63, plncFll_CTXM3_218-74, plncFll_CTXM3_220-59, and plncFll_CTXM3_221-24 have been uploaded to the NCBI GenBank database with the following accession numbers CP173724, CP173730, CP173733, and CP173739, respectively.

## Results

### Information on *K. pneumoniae* isolates with peculiar resistance phenotype

A total of 20 K*. pneumoniae* strains with peculiar resistance phenotype were collected and analyzed. Ten different sequence types (ST) were identified (**Table 1**). The most frequently detected ST was ST25, accounting for 20% (4/20), followed by ST967 at 15% (3/20). The sequence types ST65, ST133, and ST48 each represented 10% (2/20) of the isolates, while ST353, ST628, ST753, ST792, and ST254 were represented by a single strain. Two strains could not be assigned to any known ST. All strains exhibited resistance to FEP, with MIC values ranging from 32 to 512 µg/mL and a MIC_50_ of 128 µg/mL. In contrast, none of the strains were resistant to CAZ, with MIC values ranging from 2 to 8 µg/mL and a MIC_50_ of 8 µg/mL. Sensitivity to the carbapenems IPM, ETP and the aminoglycoside AMK was uniformly observed across all strains. The sensitivity rates for FOX and the β-lactam/β-lactamase inhibitor combination TZP were 95% and 85%, respectively. In contrast, sensitivity rates for AMC and SXT were both 40%, while the sensitivity rate for the quinolone drug LVX was notably low at 5%. All strains demonstrated resistance to CRO, CXM, and CXA (**Supplementary Table S3**).

**Table 1:** The genomic characteristics of *K. pneumoniae* isolates with a peculiar resistance phenotype.

Isolate	ST ^a^	Specimen Type ^b^	Resistance Genes ^c^		Plasmid Replicon-types ^d^	Plasmid Conjugation Experiments ^e^
			Key Resistance Gene(s)	Other Resistance Genes		
217-59	–	sp	*bla*_CTX-M-3_	*bla*_TEM-1_, *bla*_SHV-61_, *oqxA6*, *oqxB11*, *tet(A)*, *floR*, *mph(A)*, *aadA16*, *dfrA27*, *aac(6’)-Ib-D181Y*, *qnrB91*, *qnrS1*, *sul1*, *fosA*, *arr-3*	IncFII	Failure
217-63	25	sp	*bla*_OXA-10_, *bla*_CTX-M-14_	*bla*_SHV-110_, *oqxB17*, *oqxA7*, *qnrS1*, *bla*_LAP-2_, *floR*, *tet(A)*, *aph(6)-Id*, *aph(3’’)-Ib*, *cmlA5*, *aadA1*, *dfrA14*, *sul2*, *fosA6*	IncFIB (2), IncI1, IncFII	Success
217-89	48	sp	*bla*_CTX-M-3_	*bla*_TEM-1_, *bla*_SHV-172_, *mph(A)*, *sul1*, *aadA2*, *dfrA12*, *fosA*, *qnrS1*, *aph(3’)-Ia*	IncFII (2), IncFIB	Success
218-12	48	sp	*bla*_CTX-M-3_	*bla*_TEM-1_, *bla*_SHV-172_, *fosA*, *dfrA12*, *aadA2*, *sul1*, *mph(A)*, *qnrS1*, *aph(3’)-Ia*	IncFII (2), IncFIB	Success
218-13	25	sp	*bla*_OXA-10_, *bla*_CTX-M-14_	*bla*_SHV-110_, *oqxB17*, *oqxA7*, *qnrS1*, *bla*_LAP-2_, *floR*, *tet(A)*, *aph(6)-Id*, *aph(3’’)-Ib*, *cmlA5*, *aadA1*, *dfrA14*, *sul2*, *fosA6*	IncFIB (2), IncI1, IncFII	Success
218-19	25	cv	*bla*_CTX-M-14_	*bla*_SHV-110_, *oqxB17*, *oqxA7*, *qnrS1*, *blaLAP-2*, *aph(3’’)-Ib*, *aph(6)-Id*, *tet(A)*, *floR*, *sul2*, *fosA6*	IncFIB (2), IncI1, IncFII	Success
218-42	628	wd	*bla*_CTX-M-3_	*bla*_TEM-1_, *bla*_SHV-110_, *oqxA8*, *oqxB13*, *tet(A)*, *floR*, *mph(A)*, *aac(6’)-Ib-D181Y*, *dfrA27*, *aadA16*, *aph(6)-Id*, *aph(3’’)-Ib*, *sul2*, *qnrB91*, *fosA6*, *qnrS1*, *aac(3)-IId*, *aph(3’)-Ia*, *sul1*, *arr-3*	IncFII	Failure
218-74	65	sp	*bla*_CTX-M-3_	*bla*_SHV-11_, *tet(A)*, *floR*, *sul2*, *aph(3’’)-Ib*, *aph(6)-Id*, *qnrS1*, *fosA6*, *oqxB6*, *oqxA*	IncFII, IncHI1B, IncFIB	Success
219-10	25	ab	*bla*_CTX-M-3_	*bla*_TEM-1_, *bla*_SHV-110_, *floR*, *tet(A)*, *mef(B)*, *sul3*, *aadA1*, *cmlA1*, *aadA2*, *dfrA12*, *aac(6’)-Ib-D181Y*, *dfrA27*, *aadA16*, *sul1*, *mph(A)*, *sul2*, *aph(3’’)-Ib*, *aph(6)-Id*, *aac(3)-IId*, *qnrS1*, *oqxA7*, *oqxB17*, *aph(3’)-Ia*, *fosA6*, *arr-3*	IncFIB, IncFII (2)	Failure
219-51	2154	ps	*bla*_CTX-M-3_	*bla*_TEM-1_, *bla*_SHV-187_, *mph(A)*, *sul1*, *qnrB91*, *sul1*, *aadA16*, *dfrA27*, *aac(6’)-Ib-D181Y*, *tet(A)*, *floR*, *sul2*, *aph(3’’)-Ib*, *aph(6)-Id*, *qnrS1*, *aac(3)-IId*, *aph(3’)-Ia*, *fosA6*, *oqxA9*, *oqxB18*, *arr-3*	IncFII	Failure
219-96	133	sp	*bla*_CTX-M-14_	*bla*_SHV-75_, *oqxB4*, *oqxA10*, *fosA6*, *qnrS1*	IncFIB, IncFII (2)	Success
220-4	753	sp	*bla*_CTX-M-3_, *bla*_OXA-10_	*bla*_TEM-1_, *bla*_SHV-110_, *fosA*, *mph(A)*, *sul1*, *qnrB91*, *sul1*, *aadA16*, *dfrA27*, *aac(6’)-Ib-D181Y*, *blaLAP-2*, *qnrS1*, *floR*, *tet(A)*, *dfrA14*, *aadA1*, *cmlA5*, *aph(6)-Id*, *aph(3’’)-Ib*, *sul2*, *oqxA11*, *oqxB20*, *aph(3’)-Ia*, *arr-3*	IncFII (2), IncFIB	Failure
220-59	792	sp	*bla*_CTX-M-3_	*bla*_TEM-1_, *bla*_SHV-187_, *oqxB19*, *oqxA9*, *fosA*, *qnrS1*	IncFII	Success
220-61	133	sp	*bla*_CTX-M-14_	*bla*_SHV-75_, *fosA6*, *oqxA10*, *oqxB4*	IncR, IncFII	Success
220-98	65	sp	*bla*_CTX-M-3_	*bla*_SHV-11_, *floR*, *tet(A)*, *aph(6)-Id*, *aph(3’’)-Ib*, *sul2*, *qnrS1*, *fosA6*, *oqxB6*, *oqxA*	IncFII, IncHI1B, IncFIB	Success
221-5	–	cv	*bla*_CTX-M-14_	*bla*_SHV-75_, *oqxB4*, *oqxA10*, *tet(A)*, *dfrA1*, *qnrS1*, *bla*_LAP-2_, *tet(D)*, *fosA6*	IncFII, IncFIB	Failure
221-22	967	bl	*bla*_CTX-M-3_	*bla*_TEM-1_, *bla*_SHV-27_, *fosA*, *oqxB21*, *oqxA6*, *mph(A)*, *sul1*, *qnrB91*, *sul1*, *aadA16*, *dfrA27*, *aac(6’)-Ib-D181Y*, *floR*, *tet(A)*, *aph(6)-Id*, *aph(3’’)-Ib*, *sul2*, *qnrS1*, *aac(3)-IId*, *aph(3’)-Ia*, *arr-3*	IncFII (2), IncFIB	Failure
221-24	967	cv	*bla*_CTX-M-3_	*bla*_TEM-1_, *bla*_SHV-27_, *fosA*, *oqxA6*, *oqxB21*, *mph(A)*, *sul1*(2), *qnrB91*, *aadA16*, *dfrA27*, *aac(6’)-Ib-D181Y*, *floR*, *tet(A)*, *aph(6)-Id*, *aph(3’’)-Ib*, *sul2*, *qnrS1*, *aac(3)-IId*, *aph(3’)-Ia*, *arr-3*	IncFII (2), IncFIB	Failure
221-29	353	wd	*bla*_CTX-M-3_	*bla*_TEM-1_, *bla*_SHV-187_, *mph(A)*, *qnrB91*, *sul1*(2), *aadA16*, *dfrA27*, *aac(6’)-Ib-D181Y*, *floR*, *tet(A)*, *aph(6)-Id*, *aph(3’’)-Ib*, *sul2*, *aac(3)-IId*, *qnrS1*, *aph(3’)-Ia*, *fosA6*, *oqxA10*, *oqxB20*, *arr-3*	IncFII, IncFIB	Failure
221-35	967	sp	*bla*_CTX-M-3_	*bla*_SHV-27_, *oqxB21*, *oqxA6*, *fosA*, *mph(A)*, *sul1*, *aadA16*, *dfrA27*, *aac(6’)-Ib-D181Y*, *tet(A)*, *floR*, *aph(6)-Id*, *aph(3’’)-Ib*, *sul2*, *qnrS1*, *aac(3)-IId*, *aph(3’)-Ia*, *arr-3*	IncFIB (2), IncFII (2), IncR	Failure

a) “-” indicates that the ST type remains unidentified.

b) Specimen types: sp (sputum), cv (clean-catch midstream urine), wd (wound), ab (abscess), ps (pleural fluid), bl (blood).

c) Key Resistance Genes represent β-lactamases associated with the peculiar resistance phenotype.

d) The plasmid replicons identified in the strain are listed, with the number in parentheses indicating the count of replicons; data for the COL plasmid is not presented.

e) “Success” is defined as the successful acquisition of the transconjugant, while “failure” indicates that the transconjugant was not obtained. The primary reason for “failure” is the presence of the *arr-3* gene in the donor, which confers resistance to RIF.

### *bla_CTX-M-3_* or *bla_OXA-10_* contribute to the development of K. pneumoniae isolates with peculiar resistance phenotype

The *bla*_CTX-M-3_ gene was detected in 14 isolates, while *bla*_CTX-M-14_ was identified in 6 isolates and *bla*_OXA-10_ in 3 isolates (**Table 1**). The *bla*_TEM-1_ gene was identified in 11 isolates, all of which co-harbored *bla*_CTX-M-3_. Transconjugants were successfully obtained from ten isolates through plasmid conjugation experiments. PCR analysis confirmed the presence of *bla*_CTX-M-3_ gene in five transconjugants, *bla*_OXA-10_ in two, and *bla*_CTX-M-14_ in three. These transconjugants displayed distinct resistance phenotypes (**Supplementary Table S3**). The MIC of FEP in transconjugants carrying *bla*_CTX-M-3_ and *bla*_OXA-10_ was 16 to 32 times higher than that of CAZ, whereas in transconjugants carrying *bla*_CTX-M-14_ the FEP MIC was only 1 to 2 times higher. By integrating the susceptibility profiles of the donor strain and the recipient strain C600, it was demonstrated that *bla*_CTX-M-3_ and *bla*_OXA-10_ contribute to the development of this peculiar resistance phenotype in *K. pneumoniae*. Additionally, nine isolates were found to harbor the *arr-3* gene, which mediates resistance to RIF and was identified as the primary factor causing the failure of the plasmid conjugation experiments. *bla*_CTX-M-3_-positive isolates consistently carried multiple resistance genes, with the total number ranging from 7 to 22. Notably, most strains harbored more than 15 resistance genes. These genes conferred resistance to multiple drug classes, including β-lactams, quinolones, tetracyclines, aminoglycosides, and macrolides.

### The location of bla_CTX-M-3_ and bla_OXA-10_ gene

The IncFII plasmid replicon was detected in all isolates, while the IncFIB plasmid replicon was identified in 15 of 20 isolates (75%). Additional plasmid replicons included IncI1 (3/20, 15%), IncHI1B (2/20, 10%), and IncR (2/20, 10%). The PCR test of the conjugate confirmed that both the *bla*_CTX-M-3_ and *bla*_OXA-10_ genes were carried by the IncFII plasmid (**Supplementary Table S2**). To further investigate the genomic context, five representative strains (217-63, 218-74, 219-96, 220-59, and 221-24) underwent third generation long-read sequencing. The analysis revealed that the *bla*_CTX-M-3_ gene in strains 219-96, 220-59, and 221-24, as well as the *bla*_OXA-10_ gene in strain 217-63, were all located on the IncFII plasmid. These findings corroborate the results of the conjugation experiments (**Table 2**).

**Table 2:** Information about plasmid carrying *bla*_CTX-M-3_ or *bla*_OXA-10_ or *bla*_CTX-M-14_^a^.

Isolate	ST	Inc ^b^	Plasmid length (bp)	Resistance genes carried by this plasmid	Resistance genes carried by isolate chromosomes	Other plasmids and the resistance genes
217-63	25	IncFII IncFIB	261642	*bla*_OXA-10_, *bla*_LAP-2_, *sul2*, *aph(3’’)-Ib*, *aph(6)-Id*, *tet(A)*, *floR*, *cmlA5*, *aadA1*, *dfrA14*, *qnrS1*	*bla*_SHV-110_, *oqxB17*, *oqxA7*, *fosA6*	IncI1: *sul2*, *bla*_CTX-M-14_
218-74	65	IncFII	119574	*bla*_CTX-M-3_, *sul2*, *aph(3’’)-Ib*, *aph(6)-Id*, *tet(A)*, *floR*, *qnrS1*	*bla*_SHV-11_, *oqxB6*, *oqxA*, *fosA6*	/
219-96	133	/	/	*/*	*bla*_CTX-M-14_ (2), *bla*_SHV-75_, *qnrS1*, *oqxB4*, *oqxA10*, *fosA6*	IncFII: *bla*_CTX-M-14_, *qnrS1*
220-59	792	IncFII	71104	*bla*_CTX-M-3_, *bla*_TEM-1_, *qnrS1*	*bla*_SHV-187_, *fosA*, *oqxB19*, *oqxA9*	/
221-24	967	IncFII	88594	*bla*_CTX-M-3_, *bla*_TEM-1_, *sul2*, *aph(3’’)-Ib*, *aph(6)-Id*, *aph(3’)-Ia*, *aac(3)-IId*, *aac(6’)-Ib-D181Y*, *arr-3*, *dfrA27*, *aadA16*, *sul1* (2), *qnrB91*, *mph(A)*, *tet(A)*, *floR*, *qnrS1*	*bla*_SHV-27_, *oqxB21*, *oqxA6*, *fosA*, *sul2*	/

a) “/” means not applicable or blank.

b) Plasmid incompatibility group carrying *bla*_CTX-M-3_ or *bla*_OXA-10_ genes. The plasmids in isolates 217-63, 218-74, 220-59, and 221–24 were designated as plncFll+FIB_OXA10-217-63, lncFll_CTXM3_218-74, plncFll_CTXM3_220-59, and plncFll_CTXM3_221-24, respectively.

**Figure 1:**
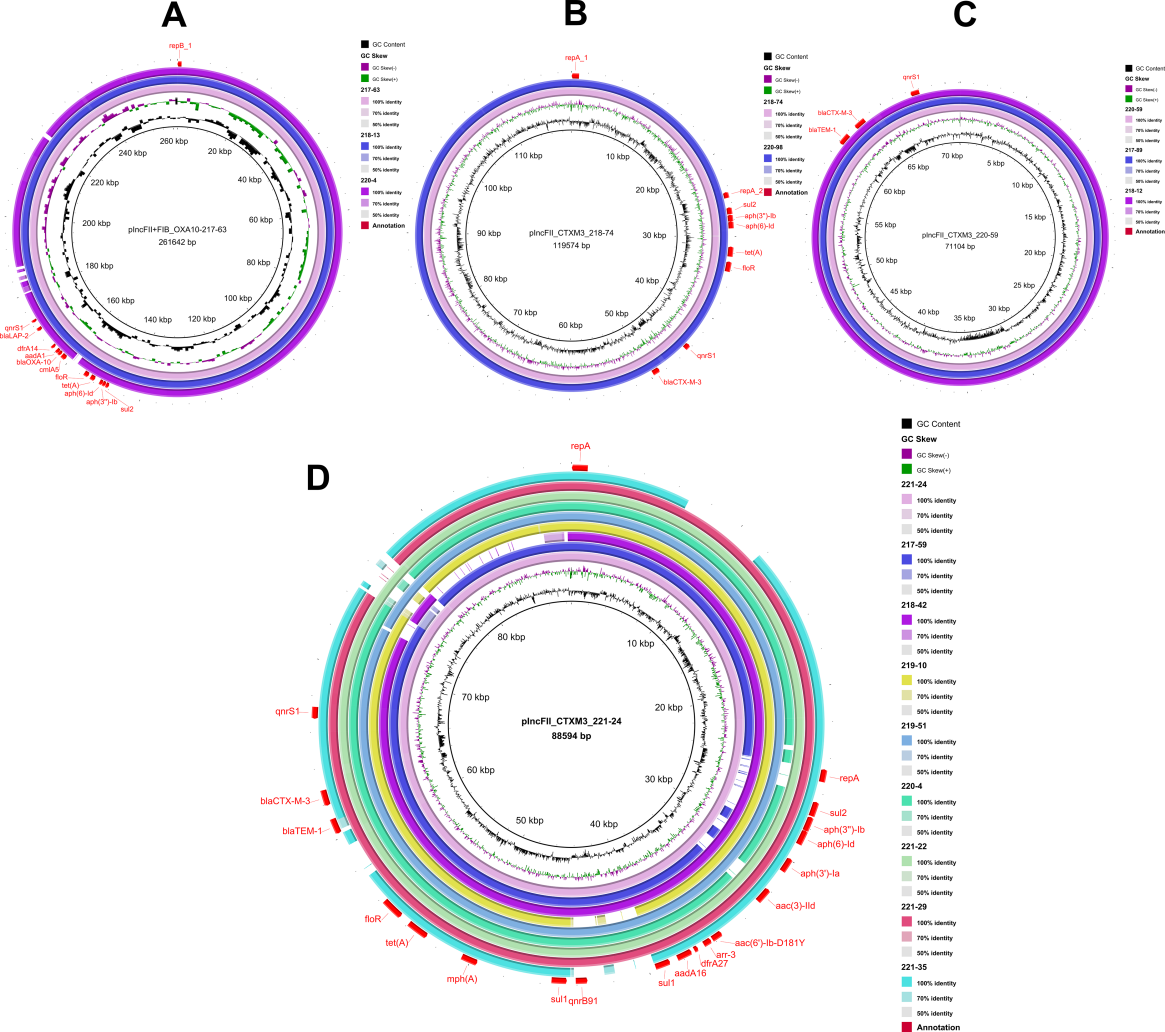
Plasmid backbone comparison. Using the corresponding plasmid sequence as a reference, the inner circle represents the GC content, while the second circle illustrates the GC skewness. The third circle depicts the sequence on the second-generation sequencing map of the isolate that carries the corresponding plasmid. Additional circles correspond to different isolates, which are identified by their logos located in the upper right corner of the figure. Annotations in the outermost circle highlight the locations of plasmid replicons and resistance genes, which are indicated in red font. **(A)**, plncFll+FIB_OXA10-217-63; **(B)**, plncFll_CTXM3_218-74; **(C)**, plncFll_CTXM3_220-59; **(D)**, plncFll_CTXM3_221-24.

Using the plasmid sequence identified in this study as references, circular plasmid maps were constructed using BRIG (**Figure 1**). This analysis revealed three distinct plasmid backbones carrying the *bla*_CTX-M-3_ gene, each characterized by unique combinations of resistance genes within their variable regions (**Figure 1B-D**). In contrast, the plasmid backbone carrying the *bla*_OXA-10_ was identical across isolates, with a largely consistent set of associated resistance genes (**Figure 1A**).

The fusion plasmid in isolate 217-63, containing both the IncFII and IncFIB elements and carrying the *bla*_OXA-10_, was designated plncFll+FIB_OXA10-217-63. This plasmid had a total length of 261,642 bp and carried ten additional resistance genes: *bla*_LAP-2_, *sul2*, *aph(3’’-Ib)*, *aph(6)-Id*, *tet(A)*, *floR*, *cmlA5*, *aadA1*, *dfrA14*, and *qnrS1* (**Figure 1A**). Comparative BRIG analysis of other *bla*_OXA-10_-positive isolates revealed that isolates 218–13 and 220–4 shared nearly identical plasmid backbones and resistance gene profiles with pIncFII+FIB_OXA10-217-63, suggesting a conserved genetic architecture among these plasmids.

The IncFII plasmid in isolate 218-74, carrying *bla*_CTX-M-3,_ was designated as plncFll_CTXM3_218-74. This plasmid spaned 119,574 bp and harbored additional resistance genes, including *sul2*, *aph(3’’)-Ib*, *aph(6)-Id*, *tet(A)*, *floR*, and *qnrS1* (**Figure 1B**). In isolate 220-59, the IncFII plasmid carrying *bla*_CTX-M-3_ was named plncFll_CTXM3_220-59, with a total length of 71,104 bp. This plasmid also contained *bla*_TEM-1_ and *qnrS1* (**Figure 1C**). Similarly, the IncFII plasmid in isolate 221-24, referred to as plncFll_CTXM3_221-24, had a total length of 88,594 bp and encoded 16 additional resistance genes, including *bla*_TEM-1_, *sul2*, *aph(3’’)-Ib*, *aph(6)-Id*, *aph(3’)-Ia*, *aac(3)-IId*, *aac(6’)-Ib-D181Y*, *arr-3*, *dfrA27*, *aadA16*, *sul1* (2), *qnrB91*, *mph(A)*, *tet(A)*, *floR*, and *qnrS1* (**Figure 1D**). Notably, the plncFll_CTXM3_221–24 plasmid backbone was the most prevalent type carrying the *bla*_CTX-M-3_ gene in this study, with homologous sequences mapped in eight isolates. Comparative BRIG analysis using pIncFII_CTXM3_218-74, pIncFII_CTXM3_220-59, and pIncFII_CTXM3_221–24 as references revealed conserved plasmid architectures among *bla*_CTX-M-3_-positive isolates: (I) The plasmid from isolate 220–98 shared backbone structure and resistance gene composition with pIncFII_CTXM3_218-74; (II) Isolates 217–89 and 218–12 carried plasmids structurally aligned with pIncFII_CTXM3_220-59; (III) A cluster of eight isolates (217-59, 218-42, 219-10, 219-51, 220-4, 221-22, 221-29, 221-35) exhibited plasmids matching the genetic organization of the dominant pIncFII_CTXM3_221-24.

### The genetic environment of bla_CTX-M-3_ and bla_OXA-10_ gene

**Figure 2:**
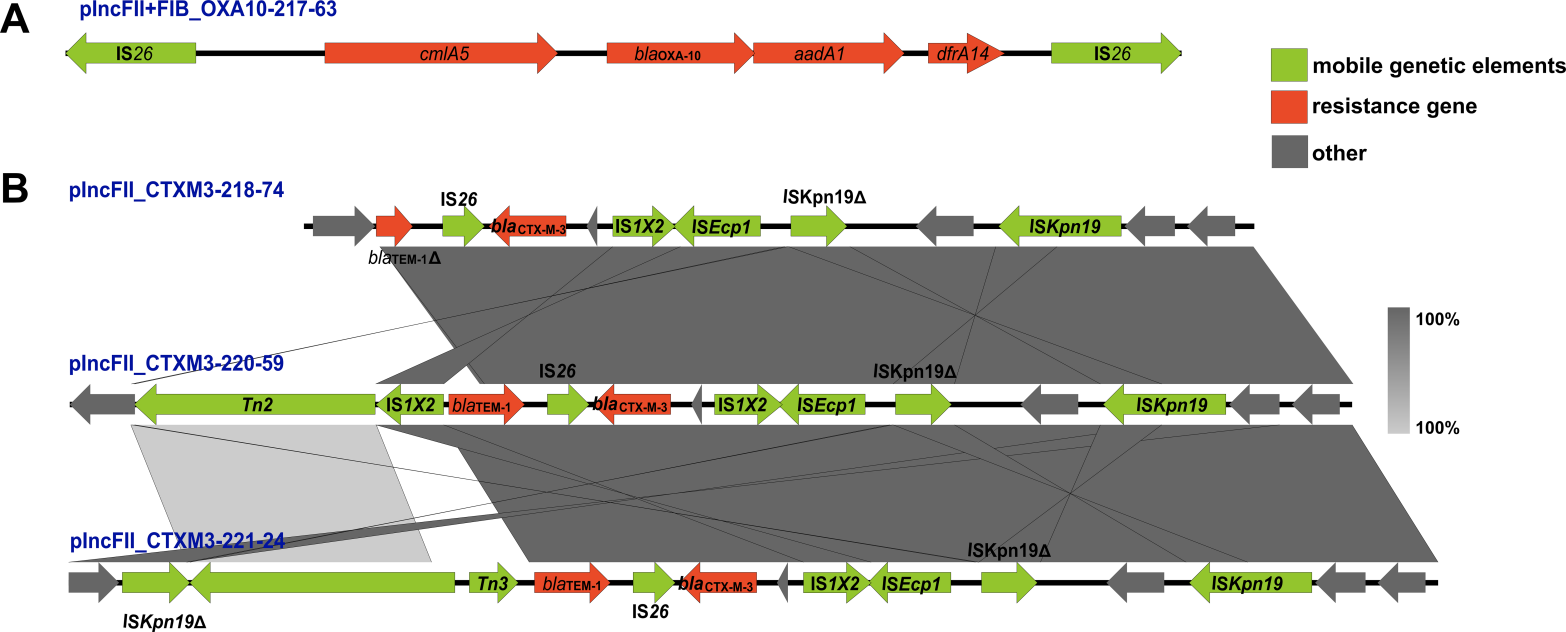
The genetic environment of *bla*_CTX-M-3_ and *bla*_OXA-10_ gene. Arrows of various colors represent distinct open reading frames (ORFs), with red indicating drug resistance genes, green denoting mobile genetic elements, and grey signifying other genes. The direction of the arrow illustrates the direction of transcription. Regions of sequence identity are highlighted by light grey shading. The symbol Δ indicates that the gene is incomplete. **(A)** depicts the genetic environment surrounding *bla*_OXA-10_ on plasmid plncFll+FIB_OXA10-217-63, while **(B)** illustrates the genetic environment surrounding *bla*_CTX-M-3_ on plasmids plncFll_CTXM3_218-74, plncFll_CTXM3_220-59, and plncFll_CTXM3_221-24.

The gene environments (**Figure 2**) of *bla*_OXA-10_ and *bla*_CTX-M-3_ were reconstructed using the extracted regions from four plasmids with known sequences, as illustrated in **Figure 1**. The genetic structure of *bla*_OXA-10_ is characterized by the arrangement “IS*26*-*cmlA5*- *bla*_OXA-10_-*aadA1*-IS*26*” (**Figure 2A**). In contrast, the *bla*_CTX-M-3_ gene was associated with a diverse array of mobile genetic elements, including IS*26*, IS*1X2*, IS*Ecp1*, IS*Kpn19*, Tn2, and Tn3. Additionally, the *bla*_TEM-1_ gene is frequently found in close proximity to *bla*_CTX-M-3_ (**Figure 2B**). Notably, the *bla*_TEM-1_ gene on plasmid pIncFII_CTXM3_218–74 is incomplete.

### Phylogenetic analysis of *bla_CTX-M-3_*-positive *K. pneumoniae* isolates

The complete sequences of 68 *bla*_CTX-M-3_-positive *K. pneumoniae* genomes were downloaded from the NCBI Pathogen Detection Database (accessed on August 20, 2024). These genomes were analyzed alongside the 14 *bla*_CTX-M-3_-positive *K. pneumoniae* isolates investigated in this study to assess their evolutionary relationships (Figure supplement 1). The figure presented key features of the isolates, including the STs, the type of plasmid Inc replicon, and associated resistance genes. The analysis revealed that *bla*_CTX-M-3_-positive *K. pneumoniae* exhibited high genetic diversity, encompassing over 40 distinct STs without a single dominant ST. Interestingly, the majority of *bla*_CTX-M-3_-positive isolates were found to harbor IncFII plasmid replicons and frequently carried TEM-type and SHV-type resistance genes.

## Discussion

The prevalence of *K. pneumoniae* isolates exhibiting peculiar resistance phenotype in this study highlights the complexity of antimicrobial resistance in this pathogen. FEP, a fourth-generation cephalosporin representative, is widely used for treating infections caused by GNB (Endimiani et al., 2008; Shoji et al., 2016). Due to its enhanced stability against hydrolysis by extended-spectrum β-lactamases (ESBLs), it typically exhibits superior *in vitro* activity compared to third-generation cephalosporins (Fung-Tomc et al., 1989; Okamoto et al., 1994). Paradoxically, our clinical observations revealed an anomalous resistance phenotype in some *K. pneumoniae* isolates, showing reduced susceptibility to FEP compared to CAZ-a phenomenon we designated as the “peculiar resistance phenotype”. The 20 K*. pneumoniae* strains analyzed displayed significant genetic diversity, with 10 distinct STs identified, including ST25 (4/20), ST967 (3/20), ST65 (2/20), ST133 (2/20), ST48 (2/20), ST353 (1/20), ST628 (1/20), ST753 (1/20), ST792 (1/20), and ST254 (1/20). Notably, several of these ST types, such as ST25, ST967, and ST65, have been previously reported in clinical isolates (Yu et al., 2018). Notably, no single ST was dominant among the isolates, suggesting that the peculiar resistance phenotype is not restricted to a specific *K. pneumoniae* clonal lineage. Phylogenetic analyzed comparing the strains in this study with those retrieved from NCBI further supported this observation.

In this study, all isolates demonstrated resistance to FEP while remaining susceptible or exhibiting intermediate susceptibility to CAZ. Among these isolates, the presence of the *bla*_CTX-M-3_ gene in 14 strains is a key contributor to high-level resistance to FEP. CTX-M-3 is a member of the CTX-M type of ESBLs, first reported in 1998 in strains of *Citrobacter freundii* and *Escherichia coli* from a Polish hospital (Gniadkowski et al., 1998). Currently, it has been identified in various *Enterobacteriaceae*, including *K. pneumoniae*, *Enterobacter cloacae*, and *Morganella morganii (* Moriguchi et al., 2007; Furlan et al., 2020; Luo et al., 2020; Souna et al., 2022). However, it appears that prior to this study, the potential of the *bla*_CTX-M-3_ gene to facilitate the emergence of peculiar resistance phenotype had not been adequately recognized. The conjugation experiments and associated data presented herein demonstrate that the IncFII plasmid carrying *bla*_CTX-M-3_ can mediate the development of higher-level peculiar resistance phenotype. In contrast, the role of *bla*_CTX-M-14_ appears to be more restricted. The mechanisms driving resistance in *P. aeruginosa* differed markedly from those in *K. pneumoniae*. In *P. aeruginosa*, the mexXY-OprM efflux system plays a central role, supplemented by various β-lactamases, including *bla*_OXA-1_, *bla*_OXA-10_, *bla*_OXA-31_, *bla*_OXA-35_, and *bla*_PSE-1_ *(* Aubert et al., 2001; Hocquet et al., 2006; Campo Esquisabel et al., 2011). Notably, *bla*_OXA-10_ was identified in only a limited number of strains in this study.

Mobile genetic elements, including plasmids, transposons, and integrons, are crucial drivers of the horizontal transfer of antibiotic resistance genes among bacterial strains (Su et al., 2018; Tran et al., 2021). The *bla*_CTX-M-3_ gene is predominantly located on an IncFII plasmid in this study. This plasmid is recognized for its high mobility and capacity to transfer resistance genes across various bacterial species (De Koster et al., 2022). In addition to *bla*_CTX-M-3_, the IncFII plasmid harbors multiple other resistance determinants, thereby contributing to multidrug resistance in the host bacterium. The presence of such a plasmid significantly enhances the adaptability and survival of resistant strains, rendering them difficult to treat with commonly used antibiotics. This study identified three distinct IncFII plasmid backbones carrying *bla*_CTX-M-3_, represented by pIncFII_CTXM3_218-74, pIncFII_CTXM3_220-59, and pIncFII_CTXM3_221-24. Among these, pIncFII_CTXM3_221–24 was the most prevalent. In addition to *bla*_CTX-M-3_, pIncFII_CTXM3_221–24 carries a variety of other resistance genes, including *bla*_TEM-1_, *sul2*, *aph(3’’)-Ib*, *aph(6)-Id*, *aph(3’)-Ia*, *aac(3)-IId*, *aac(6’)-Ib-D181Y*, *arr-3*, *dfrA27*, *aadA16*, *sul1* (2), *qnrB91*, *mph(A)*, *tet(A)*, *floR*, and *qnrS1*. These genes confer resistance to a broad range of antibiotics, including sulfonamides, aminoglycosides, tetracyclines, and quinolones, further complicating treatment strategies for infections caused by these strains. Given the potential of plasmids for widespread dissemination and their role in the emergence of resistant pathogens, it is essential to closely monitor the spread of IncFII plasmids in clinical settings to mitigate the impact of antibiotic resistance. Furthermore, the *bla*_CTX-M-3_ gene was frequently associated with mobile genetic elements such as IS*26*, IS*Ecp1*, and Tn*3*, which are well-documented facilitators of gene mobilization and horizontal transfer (Bevan et al., 2017; Partridge et al., 2018). The proximity of these elements to *bla*_CTX-M-3_ supported the hypothesis that these genes could be rapidly disseminated within bacterial populations, significantly contributing to the spread of resistance. Moreover, the co-location of *bla*_CTX-M-3_ with other resistance genes, including *bla*_TEM-1_, highlighted the complexity of resistance gene clusters in *K. pneumoniae*. This arrangement increased the potential for co-selection of resistance traits, particularly under high antibiotic pressure, where multiple resistance determinants might be selected simultaneously. In the case of *bla*_OXA-10_, the integron gene cassette array “*aadA1*-*bla*_OXA-10_-*cmlA5*-*arr2*-*dfrA14*” had been identified in various plasmids associated with several *Enterobacteriaceae* species, indicating its widespread dissemination (Arpin et al., 2012; Suzuki et al., 2019). Similar resistance genes were present around *bla*_OXA-10_ in this study. The presence of the composite transposon structure “IS*26*-*cmlA5*-*bla*_OXA-10_-*aadA1*-IS*26*” in this study suggested that this gene resided within a relatively stable genetic framework. This stability likely enhanced its persistence within the *Klebsiella pneumoniae* and facilitated its propagation (Harmer and Hall, 2016).

Our study provided valuable insights into the molecular mechanisms underlying the peculiar resistance phenotype observed in *K. pneumoniae* strains. Clinicians should be aware that certain *K. pneumoniae* strains may harbor β-lactamase genes capable of conferring differential resistance to specific cephalosporins, posing potential challenges in treatment decision-making, especially in the context of empiric therapy. Thankfully, while resistance to β-lactam antibiotics, such as FEP, the strains retained susceptibility to carbapenems (IPM and ETP) and aminoglycosides (AMK). This susceptibility pattern of resistance underscored the critical role of carbapenems as the last-resort therapy for MDR Gram-negative infections (Liu et al., 2022; Wu et al., 2022). In summary, this study enhanced our understanding of the complex resistance mechanisms in *K. pneumoniae*, with a specific emphasis on the pivotal roles of *bla*_CTX-M-3_ and *bla*_OXA-10_. The emergence of peculiar resistance phenotype underscores the urgent need for ongoing monitoring and the development of novel therapeutic strategies to address the growing threat posed by multidrug-resistant *K. pneumoniae*.

## Conclusion

This study identified a peculiar resistance phenotype in *K. pneumoniae*, characterized by reduced susceptibility to cefepime compared to ceftazidime, accompanied by a diverse range of STs. The absence of a dominant ST indicated that that this resistance phenotype was widely distributed across diverse clonal populations. Further analysis revealed that the primary driver of this phenotype is the presence of the IncFII plasmid carrying the *bla*_CTX-M-3_ gene. This plasmid not only harbored multiple additional resistant genes but also demonstrated high conjugation and transferability, facilitating the dissemination of resistant genes. Moreover, some strains possessed an IncFII/IncFIB fusion plasmid that contains the *bla*_OXA-10_ gene, which further enhances resistance. This resistance mechanism in *K. pneumoniae* shows significant differences compared to *P. aeruginosa*. These findings underscore the critical role of specific plasmids in the development of the resistance phenotype in *K. pneumoniae*, which could complicate the treatment of infections. Clinicians may need to consider alternative antibiotics or combination therapies, and surveillance for such resistant strains should be prioritized to guide effective treatment.

## Data Availability

The datasets presented in this study can be found in online repositories. The names of the repository/repositories and accession number(s) can be found in the article/**Supplementary Material**.

## References

[B1] Alikhan N. F. Petty N. K. Ben Zakour N. L. Beatson S. A. (2011). BLAST Ring Image Generator (BRIG): simple prokaryote genome comparisons. BMC Genomics 12, 402. doi: 10.1186/1471-2164-12-402 21824423 PMC3163573

[B2] Arpin C. Thabet L. Yassine H. Messadi A. A. Boukadida J. Dubois V.. (2012). Evolution of an incompatibility group IncA/C plasmid harboring blaCMY-16 and qnrA6 genes and its transfer through three clones of Providencia stuartii during a two-year outbreak in a Tunisian burn unit. Antimicrob. Agents Chemother. 56, 1342–1349. doi: 10.1128/AAC.05267-11 22155825 PMC3294913

[B3] Aubert D. Poirel L. Chevalier J. Leotard S. Pages J. M. Nordmann P. (2001). Oxacillinase-mediated resistance to cefepime and susceptibility to ceftazidime in Pseudomonas aeruginosa. Antimicrob. Agents Chemother. 45, 1615–1620. doi: 10.1128/AAC.45.6.1615-1620.2001 11353602 PMC90522

[B4] Bankevich A. Nurk S. Antipov D. Gurevich A. A. Dvorkin M. Kulikov A. S.. (2012). SPAdes: a new genome assembly algorithm and its applications to single-cell sequencing. J. Comput. Biol. 19, 455–477. doi: 10.1089/cmb.2012.0021 22506599 PMC3342519

[B5] Bevan E. R. Jones A. M. Hawkey P. M. (2017). Global epidemiology of CTX-M beta-lactamases: temporal and geographical shifts in genotype. J. Antimicrob. Chemother. 72, 2145–2155. doi: 10.1093/jac/dkx146 28541467

[B6] Bortolaia V. Kaas R. S. Ruppe E. Roberts M. C. Schwarz S. Cattoir V.. (2020). ResFinder 4.0 for predictions of phenotypes from genotypes. J. Antimicrob. Chemother. 75, 3491–3500. doi: 10.1093/jac/dkaa345 32780112 PMC7662176

[B7] Campo Esquisabel A. B. Rodriguez M. C. Campo-Sosa A. O. Rodriguez C. Martinez-Martinez L. (2011). Mechanisms of resistance in clinical isolates of Pseudomonas aeruginosa less susceptible to cefepime than to ceftazidime. Clin. Microbiol. Infect. 17, 1817–1822. doi: 10.1111/j.1469-0691.2011.03530.x 21599797

[B8] De Koster S. Rodriguez Ruiz J. P. Rajakani S. G. Lammens C. Glupczynski Y. Goossens H.. (2022). Diversity in the Characteristics of Klebsiella pneumoniae ST101 of Human, Environmental, and Animal Origin. Front. Microbiol. 13, 838207. doi: 10.3389/fmicb.2022.838207 35222344 PMC8866942

[B9] Endimiani A. Perez F. Bonomo R. A. (2008). Cefepime: a reappraisal in an era of increasing antimicrobial resistance. Expert Rev. Anti Infect. Ther. 6, 805–824. doi: 10.1586/14787210.6.6.805 19053894 PMC2633657

[B10] Fung-Tomc J. Dougherty T. J. DeOrio F. J. Simich-Jacobson V. Kessler R. E. (1989). Activity of cefepime against ceftazidime- and cefotaxime-resistant gram-negative bacteria and its relationship to beta-lactamase levels. Antimicrob. Agents Chemother. 33, 498–502. doi: 10.1128/AAC.33.4.498 2499250 PMC172467

[B11] Furlan J. P. R. Lopes R. Gonzalez I. H. L. Ramos P. L. von Zeska Kress M. R. Stehling E. G. (2020). Hypermucoviscous/hypervirulent and extensively drug-resistant QnrB2-, QnrS1-, and CTX-M-3-coproducing Klebsiella pneumoniae ST2121 isolated from an infected elephant (Loxodonta africana). Vet. Microbiol. 251, 108909. doi: 10.1016/j.vetmic.2020.108909 33176213

[B12] Gniadkowski M. Schneider I. Palucha A. Jungwirth R. Mikiewicz B. Bauernfeind A. (1998). Cefotaxime-resistant Enterobacteriaceae isolates from a hospital in Warsaw, Poland: identification of a new CTX-M-3 cefotaxime-hydrolyzing beta-lactamase that is closely related to the CTX-M-1/MEN-1 enzyme. Antimicrob. Agents Chemother. 42, 827–832. doi: 10.1128/AAC.42.4.827 9559791 PMC105550

[B13] Harmer C. J. Hall R. M. (2016). IS26-Mediated Formation of Transposons Carrying Antibiotic Resistance Genes. mSphere 1 (2), e00038-16. doi: 10.1128/mSphere.00038-16 PMC4894685 27303727

[B14] Hocquet D. Nordmann P. El Garch F. Cabanne L. Plesiat P. (2006). Involvement of the MexXY-OprM efflux system in emergence of cefepime resistance in clinical strains of Pseudomonas aeruginosa. Antimicrob. Agents Chemother. 50, 1347–1351. doi: 10.1128/AAC.50.4.1347-1351.2006 16569851 PMC1426951

[B15] Liu W. Tang J. W. Lyu J. W. Wang J. J. Pan Y. C. Shi X. Y.. (2022). Discrimination between Carbapenem-Resistant and Carbapenem-Sensitive Klebsiella pneumoniae Strains through Computational Analysis of Surface-Enhanced Raman Spectra: a Pilot Study. Microbiol. Spectr. 10, e0240921. doi: 10.1128/spectrum.02409-21 35107359 PMC8809336

[B16] Luo X. Zhai Y. He D. Cui X. Yang Y. Yuan L.. (2020). Molecular characterization of a novel bla (CTX-M-3)-carrying Tn6741 transposon in Morganella morganii isolated from swine. J. Med. Microbiol. 69, 1089–1094. doi: 10.1099/jmm.0.001235 32692646

[B17] Moriguchi N. Itahashi Y. Tabata N. Yamazumi T. Furuta I. Shibata N.. (2007). Outbreak of CTX-M-3-type extended-spectrum beta-lactamase-producing Enterobacter cloacae in a pediatric ward. J. Infect. Chemother. 13, 263–266. doi: 10.1007/s10156-007-0526-7 17721690

[B18] Munoz-Price L. S. Poirel L. Bonomo R. A. Schwaber M. J. Daikos G. L. Cormican M.. (2013). Clinical epidemiology of the global expansion of Klebsiella pneumoniae carbapenemases. Lancet Infect. Dis. 13, 785–796. doi: 10.1016/S1473-3099(13)70190-7 23969216 PMC4673667

[B19] Nguyen H. M. Shier K. L. Graber C. J. (2014). Determining a clinical framework for use of cefepime and beta-lactam/beta-lactamase inhibitors in the treatment of infections caused by extended-spectrum-beta-lactamase-producing Enterobacteriaceae. J. Antimicrob. Chemother. 69, 871–880. doi: 10.1093/jac/dkt450 24265230

[B20] Okamoto M. P. Nakahiro R. K. Chin A. Bedikian A. Gill M. A. (1994). Cefepime: a new fourth-generation cephalosporin. Am. J. Hosp Pharm. 51, 463–477. doi: 10.1093/ajhp/51.4.463 8017411

[B21] Partridge S. R. Kwong S. M. Firth N. Jensen S. O. (2018). Mobile Genetic Elements Associated with Antimicrobial Resistance. Clin. Microbiol. Rev. 31 (4), e00088-17. doi: 10.1128/CMR.00088-17 30068738 PMC6148190

[B22] Rodriguez-Bano J. Gutierrez-Gutierrez B. Machuca I. Pascual A. (2018). Treatment of Infections Caused by Extended-Spectrum-Beta-Lactamase-, AmpC-, and Carbapenemase-Producing Enterobacteriaceae. Clin. Microbiol. Rev. 31 (2), e00079-17. doi: 10.1128/CMR.00079-17 29444952 PMC5967687

[B23] Seemann T. (2014). Prokka: rapid prokaryotic genome annotation. Bioinformatics 30, 2068–2069. doi: 10.1093/bioinformatics/btu153 24642063

[B24] Shoji K. Bradley J. S. Reed M. D. van den Anker J. N. Domonoske C. Capparelli E. V. (2016). Population Pharmacokinetic Assessment and Pharmacodynamic Implications of Pediatric Cefepime Dosing for Susceptible-Dose-Dependent Organisms. Antimicrob. Agents Chemother. 60, 2150–2156. doi: 10.1128/AAC.02592-15 26810655 PMC4808231

[B25] Souna D. Drissi M. Almahmoud I. Maurin M. (2022). Enterobacter cloacae Complex and CTX-M Extended-Spectrum beta-Lactamases in Algeria. Microb. Drug Resist. 28 (3), 346–354. doi: 10.1089/mdr.2020.0535 34890283

[B26] Su H. Hu X. Xu Y. Xu W. Huang X. Wen G.. (2018). Persistence and spatial variation of antibiotic resistance genes and bacterial populations change in reared shrimp in South China. Environ. Int. 119, 327–333. doi: 10.1016/j.envint.2018.07.007 29990953

[B27] Sullivan M. J. Petty N. K. Beatson S. A. (2011). Easyfig: a genome comparison visualizer. Bioinformatics 27, 1009–1010. doi: 10.1093/bioinformatics/btr039 21278367 PMC3065679

[B28] Suzuki Y. Ida M. Kubota H. Ariyoshi T. Murakami K. Kobayashi M.. (2019). Multiple beta-Lactam Resistance Gene-Carrying Plasmid Harbored by Klebsiella quasipneumoniae Isolated from Urban Sewage in Japan. mSphere 4 (5), 00391-19. doi: 10.1128/mSphere.00391-19 PMC6763765 31554719

[B29] Tran T. T. Scott A. Tien Y. C. Murray R. Boerlin P. Pearl D. L.. (2021). On-Farm Anaerobic Digestion of Dairy Manure Reduces the Abundance of Antibiotic Resistance-Associated Gene Targets and the Potential for Plasmid Transfer. Appl. Environ. Microbiol. 87, e0298020. doi: 10.1128/AEM.02980-20 33931422 PMC8231723

[B30] Wick R. R. Judd L. M. Gorrie C. L. Holt K. E. (2017). Unicycler: Resolving bacterial genome assemblies from short and long sequencing reads. PloS Comput. Biol. 13, e1005595. doi: 10.1371/journal.pcbi.1005595 28594827 PMC5481147

[B31] Wu D. Huang Y. Xiao J. Qin G. Liu H. Peng J. (2022). Risk Factors for Mortality Among Critical Acute Pancreatitis Patients with Carbapenem-Resistant Organism Infections and Drug Resistance of Causative Pathogens. Infect. Dis. Ther. 11, 1089–1101. doi: 10.1007/s40121-022-00624-w 35377132 PMC9124255

[B32] Yu F. Lv J. Niu S. Du H. Tang Y. W. Pitout J. D. D.. (2018). Multiplex PCR Analysis for Rapid Detection of Klebsiella pneumoniae Carbapenem-Resistant (Sequence Type 258 [ST258] and ST11) and Hypervirulent (ST23, ST65, ST86, and ST375) Strains, J Clin Microbiol 56 (9), 00731-18. doi: 10.1128/JCM.00731-18 PMC6113471 29925644

