# Supplementary material for: Involvement of the bla CTX-M-3 gene in emergence of a peculiar resistance phenotype in Klebsiella pneumoniae
Source: Front Cell Infect Microbiol. 2025 May 13;15:1545157. doi: 10.3389/fcimb.2025.1545157 (PMC12106457; doi:10.3389/fcimb.2025.1545157)
Supplement: Supplementary file 1 [file Table1.docx]

Supplementary Table S1 The relevant primer sequences

| CTX-M-group-1-F | CAGCGCTTTTGCCGTCTAAG |
| --- | --- |
| CTX-M-group-1-R | GGCCCATGGTTAAAAAATCACTGC |
| IncFII-F | ATGACCTGTTGTTGTCG |
| IncFII-R | TTAGGATCCTGACGTTG |
| OXA-10-F | GCTTGATCGCCCTCGATT |
| OXA-10-R | GATTTGCTCCGTGGCCGAAA |

Supplementary Table S2 Transconjugant PCR test results for *bla*_CTX-M-3_-positive or *bla*_OXA-10_-positive strains.

| **Isolate** | **CTX-M-3** | **OXA-10** | **IncFII** |
| --- | --- | --- | --- |
| C-217-63 |  | + | + |
| C-217-89 | + |  | + |
| C-218-12 | + |  | + |
| C-218-13 |  | + | + |
| C-218-74 | + |  | + |
| C-220-59 | + |  | + |
| C-220-98 | + |  | + |

Supplementary Table S3 The antibiotic susceptibility characteristics of *K. pneumoniae* isolates with special resistant phenotypes and its transconjugants

| **Isolate** | **MIC（µg/mL）** | | | | | | | | | | | | | |
| --- | --- | --- | --- | --- | --- | --- | --- | --- | --- | --- | --- | --- | --- | --- |
|  | **FEP** | **CAZ** | **AMK** | **AMC** | **ETP** | **SXT** | **TZP** | **TGC** | **CXM** | **CXA** | **CRO** | **FOX** | **IPM** | **LVX** |
| 217-59 | 32 | 4 | <=2 | 16 | <=0.12 | >=320 | <=4 | 1 | >=64 | >=64 | >=64 | <=4 | <=0.25 | 4 |
| 217-63 | 512 | 8 | <=2 | 16 | <=0.12 | >=320 | 32 | 1 | >=64 | >=64 | >=64 | <=4 | <=0.25 | 1 |
| C-217-63 | 128 | 8 | <=2 | 32 | <=0.12 | <=20 | <=4 | <=0.5 | >=64 | >=64 | >=64 | 8 | <=0.25 | 0.5 |
| 217-89 | 512 | 8 | <=2 | 16 | <=0.12 | <=20 | 8 | <=0.5 | >=64 | >=64 | >=64 | <=4 | <=0.25 | 1 |
| C-217-89 | 128 | 8 | <=2 | 16 | <=0.12 | <=20 | <=4 | <=0.5 | >=64 | >=64 | >=64 | 8 | <=0.25 | 4 |
| 218-12 | 256 | 8 | <=2 | 16 | <=0.12 | <=20 | <=4 | <=0.5 | >=64 | >=64 | >=64 | <=4 | <=0.25 | 1 |
| C-218-12 | 128 | 8 | <=2 | 16 | <=0.12 | <=20 | <=4 | <=0.5 | >=64 | >=64 | >=64 | 8 | <=0.25 | 4 |
| 218-13 | 512 | 8 | <=2 | 16 | <=0.12 | >=320 | 8 | 1 | >=64 | >=64 | >=64 | <=4 | <=0.25 | 1 |
| C-218-13 | 128 | 8 | <=2 | 16 | <=0.12 | <=20 | <=4 | <=0.5 | >=64 | >=64 | >=64 | 8 | <=0.25 | 0.5 |
| 218-19 | 32 | 2 | <=2 | 8 | <=0.12 | <=20 | 8 | 1 | >=64 | >=64 | >=64 | <=4 | <=0.25 | 1 |
| C-218-19 | 4 | 2 | <=2 | 16 | <=0.12 | <=20 | <=4 | <=0.5 | >=64 | >=64 | >=64 | 8 | <=0.25 | 0.5 |
| 218-42 | 128 | 8 | <=2 | 16 | <=0.12 | >=320 | 32 |  | >=64 | >=64 | >=64 | 8 | <=0.25 | >=8 |
| 218-74 | 128 | 4 | <=2 | 8 | <=0.12 | <=20 | <=4 | 1 | >=64 | >=64 | >=64 | <=4 | <=0.25 | 1 |
| C-218-74 | 128 | 4 | <=2 | 16 | <=0.12 | <=20 | <=4 | <=0.5 | >=64 | >=64 | >=64 | 8 | <=0.25 | 4 |
| 219-10 | 64 | 8 | <=2 | 16 | <=0.12 | >=320 | <=4 | 2 | >=64 | >=64 | >=64 | <=4 | <=0.25 | 1 |
| 219-51 | 256 | 4 | <=2 | 16 | <=0.12 | >=320 | <=4 | 2 | >=64 | >=64 | >=64 | <=4 | <=0.25 | 4 |
| 219-96 | 256 | 8 | <=2 | 16 | <=0.12 | <=20 | 16 |  | >=64 | >=64 | >=64 | 32 | <=0.25 | >=8 |
| C-219-96 | 8 | 8 | <=2 | 16 | <=0.12 | <=20 | <=4 | <=0.5 | >=64 | >=64 | >=64 | 32 | <=0.25 | 4 |
| 220-4 | 64 | 8 | <=2 | 16 | <=0.12 | >=320 | 8 | 2 | >=64 | >=64 | >=64 | <=4 | <=0.25 | 4 |
| 220-61 | 128 | 4 | <=2 | 8 | <=0.12 | <=20 | 16 |  | >=64 | >=64 | >=64 | 8 | <=0.25 | 0.5 |
| C-220-61 | 8 | 4 | <=2 | 8 | <=0.12 | <=20 | <=4 | <=0.5 | >=64 | >=64 | >=64 | 32 | <=0.25 | 0.5 |
| 220-59 | 512 | 8 | <=2 | 16 | <=0.12 | <=20 | 32 |  | >=64 | >=64 | >=64 | <=4 | <=0.25 | 4 |
| C-220-59 | 128 | 8 | <=2 | 16 | <=0.12 | <=20 | <=4 | <=0.5 | >=64 | >=64 | >=64 | 8 | <=0.25 | 4 |
| 220-98 | 64 | 8 | <=2 | 8 | <=0.12 | 80 | 8 | 2 | >=64 | >=64 | >=64 | 8 | <=0.25 | >=8 |
| C-220-98 | 64 | 4 | <=2 | 16 | <=0.12 | <=20 | <=4 | <=0.5 | >=64 | >=64 | >=64 | 8 | <=0.25 | 4 |
| 221-5 | 32 | 2 | <=2 | 8 | <=0.12 | <=20 | 8 |  | >=64 | >=64 | >=64 | <=4 | <=0.25 | 4 |
| 221-24 | 512 | 8 | <=2 | 8 | <=0.12 | >=320 | <=4 | 2 | >=64 | >=64 | >=64 | <=4 | <=0.25 | >=8 |
| 221-29 | 128 | 8 | <=2 | 16 | <=0.12 | >=320 | 8 | 2 | >=64 | >=64 | >=64 | <=4 | <=0.25 | >=8 |
| 221-22 | 32 | 8 | <=2 | 8 | <=0.12 | >=320 | <=4 | 2 | >=64 | >=64 | >=64 | <=4 | <=0.25 | >=8 |
| 221-35 | 64 | 8 | <=2 | 4 | <=0.12 | >=320 | <=4 | 2 | >=64 | >=64 | >=64 | <=4 | <=0.25 | 4 |
| C600 | 0.125 | 0.25 | <=2 | 4 | <=0.12 | <=20 | <=4 | <=0.5 | 16 | 16 | <=0.25 | 8 | <=0.25 | 0.5 |


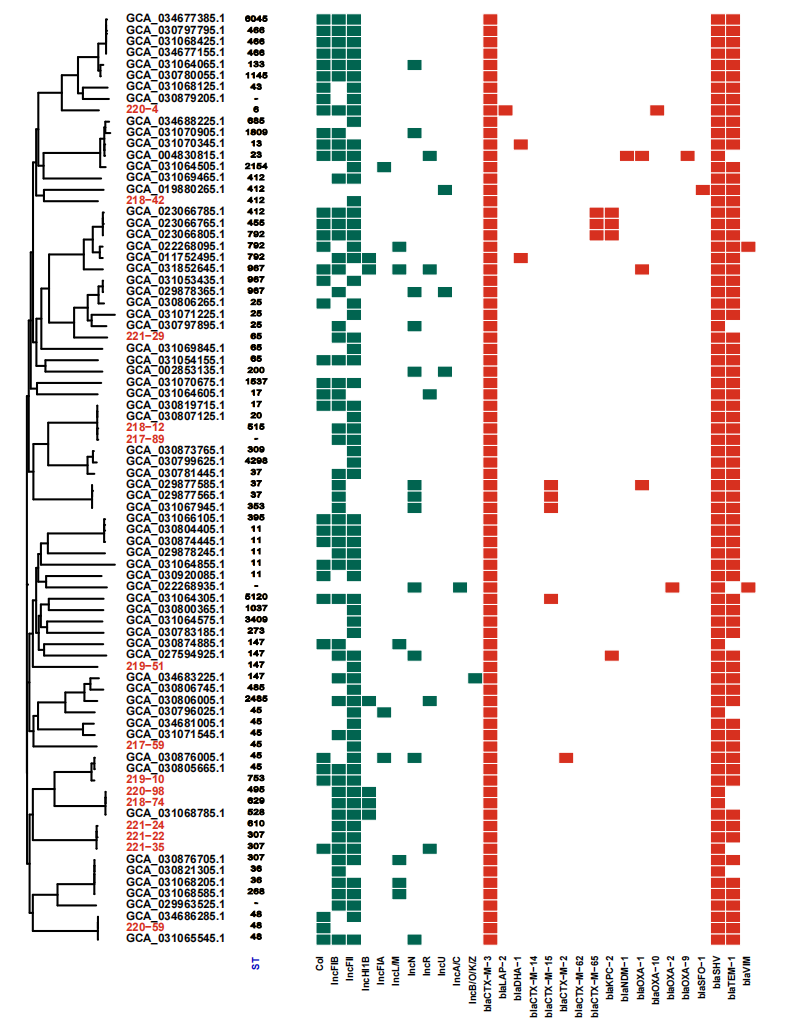


**Figure supplement 1. Phylogeny and distribution of partial antimicrobial resistance genes among *bla*_CTX-M-3_-positive *K. pneumoniae* isolates in this study, as well as those included in the NCBI database.**

The green box represents the isolate that carries the corresponding plasmid replicon, while the red box indicates the isolate that possesses the corresponding resistance gene. The empty box signifies that the respective plasmid replicon or resistance gene was not detected.
